# Stride-related rein tension patterns in walk and trot in the ridden horse

**DOI:** 10.1186/s13028-015-0182-3

**Published:** 2015-12-30

**Authors:** Agneta Egenvall, Lars Roepstorff, Marie Eisersiö, Marie Rhodin, René van Weeren

**Affiliations:** Department of Clinical Sciences, Faculty of Veterinary Medicine and Animal Husbandry, Swedish University of Agricultural Sciences, Box 7054, 750 07 Uppsala, Sweden; Unit of Equine Studies, Department of Anatomy, Physiology and Biochemistry, Faculty of Veterinary Medicine and Animal Husbandry, Swedish University of Agricultural Sciences, Box 7046, 750 07 Uppsala, Sweden; Department of Equine Sciences, Faculty of Veterinary Medicine, Utrecht University, Yalelaan 114, 3584 CM Utrecht, The Netherlands

**Keywords:** Inertial measurement unit, Rein tension, Trot, Walk, Variation

## Abstract

**Background:**

The use of tack (equipment such as saddles and reins) and especially of bits because of rein tension resulting in pressure in the mouth is questioned because of welfare concerns. We hypothesised that rein tension patterns in walk and trot reflect general gait kinematics, but are also determined by individual horse and rider effects. Six professional riders rode three familiar horses in walk and trot. Horses were equipped with rein tension meters logged by inertial measurement unit technique. Left and right rein tension data were synchronized with the gait.

**Results:**

Stride split data (0–100 %) were analysed using mixed models technique to elucidate the left/right rein and stride percentage interaction, in relation to the exercises performed. In walk, rein tension was highest at hindlimb stance. Rein tension was highest in the suspension phase at trot, and lowest during the stance phase. In rising trot there was a significant difference between the two midstance phases, but not in sitting trot. When turning in trot there was a significant statistical association with the gait pattern with the tension being highest in the inside rein when the horse was on the outer fore-inner hindlimb diagonal.

**Conclusions:**

Substantial between-rider variation was demonstrated in walk and trot and between-horse variation in walk. Biphasic rein tensions patterns during the stride were found mainly in trot.

**Electronic supplementary material:**

The online version of this article (doi:10.1186/s13028-015-0182-3) contains supplementary material, which is available to authorized users.

## Background

For most of the time since the domestication of the horse, more than five millennia ago, mankind has used the horse mainly for the capabilities of its locomotor system. It is therefore not surprising that the majority of the physical problems of the horse are orthopaedic in nature [1–3] and that the rider/trainer has an influence on the occurrence and manifestation of locomotor problems in the horse [1, 2]. It is likely that at least part of this influence is due to riding technique [1, 2].

Over time, different kinds of tack have been developed to facilitate the use of the horse, tack being equipment used on the horse to facilitate the use of it, such as saddles and reins. In many riding disciplines, reins are attached to a piece of metal that sits in the horse’s mouth—the bit. The rider uses the reins to act on the bit to provide cues to the horse to indicate desired direction, acceleration or deceleration and carriage of the head. Recently scientific interest in the use of tack and the bit in particular has surged, principally related to animal welfare. The use of bits is regularly questioned [4]. Scientific research has been made possible by the development of tools that can measure the specific effects of various elements of the tack of a horse. Rein tension has a strong influence on the effects of the bit on the sensitive tissues of the horse’s mouth and can now be measured reliably. It has recently been shown that riders influence rein tension to a large degree [5, 6].

Average rein tensions have been reported as 5.1 N at the walk, 6.3 N at the trot [7] and around 15 N at the canter [8]. Rein tension measurements at trot in unmounted horses or horses ridden with a free head and neck position showed peaks with maximal tension occurring in the second half of each diagonal stance phase [9, 10]. In sitting trot with the horse’s nose line on the vertical the largest rein tension peaks were found in the suspension phase. It should be mentioned that the latter conclusions were based on the appraisal of visual differences in a study of three horses and not on statistical analysis [10]. In a study comprising more horses, rein tension in canter was maximal just before the beginning of vertical stance, the release was closer to the suspension phase and also more marked on the outside rein (the rein facing the outside of an arena or a circle) [6]. On average, tension in the outside rein was 7 N less than in the inside rein close to the suspension phase, while at midstance tension in both reins was just over 30 N [6].

Riders are subjected to substantially different locomotion patterns in each of the three main gaits of the horse: walk trot and canter. Of these, walk and trot are symmetrical whereas canter is an asymmetrical gait. It has been shown in a treadmill study that at the slower four-beat walk the extra-sagittal movements of the saddle (i.e. yaw and roll) the rider has to accommodate are several degrees larger than at trot, as fore and hindquarter movements are not synchronous [11]. In another treadmill study it was demonstrated that in the two-beat trot there is less lateroflexion of the equine spine, leaving the sitting rider mainly subjected to vertical and longitudinal forces as the withers and croup move vertically simultaneously [12]. However, the rider can also choose to rise at trot, alternating sitting and rising on the two diagonals.

The aim of the current study was to quantify and analyse stride phase related rein tension at walk and trot. We further hypothesised that rein tension patterns would not only be influenced by the gait, but that individual horse and rider effects would exist and that the latter thus may be a contributing element to the more general rider effects as described earlier [1, 2].

## Methods

### Ethical permission

According to the Swedish legislation ethical permit was not necessary for this study.

### Riders and horses

Data were collected from six professional riders (mean ± STD height 172 ± 8 cm and weight 68 ± 12 kg), each riding three horses that were familiar to them (n = 18). The riders had regularly trained their ‘own’ horses for between 1 month and 22 years, median 24 months. All horses wore their own correctly fitting saddle and bridle with their ordinary snaffle bit. Fifteen of the snaffles had three parts, two were straight (of these one had rigid rings and one rubber ones); seven had two parts. Two of the fifteen 3-part snaffles had fixed rings, one had a small port and two of the 2-part snaffles were full-cheek. Further information on the horses and riders can be found in the study by Eisersiö et al. [13], which used the same group of riders and horses with the exception of riders 3 and 5. When asked, one rider stated left-handedness, the others said they were right-handed. Horse laterality was assessed by asking the riders to which side the horses used to bend most easily. Five horses were found to be easier to bend to the left, 11 horses were easier to bend to the right, one horse was equally easy to bend in left and right direction, and one horse was easier to bend to the right at the trot and to the left at the canter. The educational level of the horses was reported by the riders as: basic (n = 6), young horse (n = 3), medium (n = 5) and advanced (n = 4). Advanced horses had competed at Prix St. George, Intermediaire or Grand Prix level (these competition levels include several exercises of great difficulty, such as piaffe, passage and canter pirouettes that are not or rarely performed by basic horses); basic horses had entered low-level competitions only and medium horses were in between. Young horses had been ridden for less than a year and had not competed.

### Equipment

Data collection took place at each horse’s current stable in an indoor arena (n = 3 riders, 1 sand-fibre arena and two sand-wood chip arenas, the smallest 20 × 50 m and the largest 23 × 62 m), or outdoor arena (n = 3 riders, gravel-based, the smallest 23 × 62 m and the largest 40 × 80 m), depending on weather conditions. Each horse was fitted with a custom-made rein tension meter (128 Hz), measuring range 0–500 N, resolution 0.11 N, fastened on leather reins. A cable from each tension meter ran forwards along the rein and up along the side piece of the bridle (Additional file 1), passing behind the horse’s ear and ending at an Inertial Measurement Unit (IMU, x-io Technologies Limited, UK) attached right below the brow band of the bridle using Velcro. The rein tension meters, for each rein separately, were calibrated before the riding sessions started by suspending 13 known weights between 0 and 20 kg. The rein tension meter was also screened in a tensile test machine for stability and repeatability of results (one example see Additional file 2). Further details on the rein tension meter can be found elsewhere [14]. All equipment was fitted on the horse in the riding arena, which took approximately 10 min including synchronization (see below) of the equipment.

Video recordings (Canon Legria HF200, 25 Hz) were made of the entire riding session from the middle of one of the long sides of the arena. All horses were free from lameness according to the clinical judgment of a veterinarian, who visually evaluated the videos of the horses.

### Study design

Once the measuring equipment was fitted to the horse, the riders were asked to follow their normal routine with each horse for flatwork/dressage and to ride in all gaits (walk, trot and canter, the latter gait was not analysed within this study). The whole riding arena was used for the exercises and the length of the riding session was determined by the rider. More detail on the content and actual exercises performed during the riding sessions is presented in detail elsewhere [13].

### Synchronization of equipment

After the rider had mounted, and before dismounting at the end, the rein tension meter was synchronized with the video recordings by pulling on the right tension meter five times twice in a row while counting out loud in front of the camera. This procedure allowed for processing of the data from the rein tension meter with the corresponding video frames.

### Data management

One investigator (ME) scrutinized the videos and categorized the behavioural data. Further detailed information on this protocol can be found elsewhere [13]. In brief, the categories used in this study were rider’s position in the saddle (sitting, rising), corners and turns (corner left/right, turn left/right), lateral movements (half-pass to the left/right, shoulder-in left/right, leg-yield left/right) or riding in lengthening (trot with longer strides). The accuracy of the assessment and classification of the video frames by the evaluator (ME) was checked during the data analysis process by comparing to head acceleration and head angle data from the IMUs by a second person (AE). In this process the gait transitions could be traced and confirmed easily, proving correctness of gait classification. Similarly, alterations in head angles were checked and correct synchronization between data and events on video frames could be confirmed. Rein tension data were downloaded to a personal computer and handled in Matlab (MathWorks Inc., USA). Using custom-written scripts, data were split to generate half-strides (e.g. from midstance of the right forelimb to midstance of the left forelimb) based on the most vertical acceleration signal from the poll using the ‘peakfinds’ function in Matlab. Euler angles of the IMU on the croup (around the horizontal cranio-caudal axis) were used to make sure the split was made on right forelimb midstance, which in trot is the right fore/left hind diagonal and in walk at right forelimb stance. The stride-split was thus in both gaits from right forelimb mid-stance to the next right forelimb midstance. The data were graphically verified before accepting the stride-splits. Using this approach, the suspension phase in trot will start at around 25 and 75 % of the stride [15]. Time-normalised rein data (0–100 %) were constructed using stride split times. The nose angle range of motion (ROM, defined as the maximal minus the minimal nose angle that was measured) as well as whether the nose was moving backwards (in) or forwards (out) relative to the frame of the horse, was determined from Euler angles, derived from the gyroscopic inertial measurement unit signal from the head.

### Statistical modeling

The outcomes were rein tension in the left and right rein during walk and trot separately [both on short reins (long reins were defined as hanging in a loop and the horse having an unrestrained head and neck position; with short reins the rider had contact with the horse’s mouth)]. However, for each gait rein tension data on left and right reins were evaluated in the same model. Dependent data were time-normalised stride means (one series in one horse = one normalised stride of 101 data points) that each belonged to a compound category (e.g. sitting trot in half-pass to the right ridden in a turn, with baseline for corners and no lengthening). Rein tension was checked for normality, i.e. means and medians were deemed close (i.e. mean differing from median by preferably not more than 5 % of the median), the standard deviations judged as small, and skewness and kurtosis close to zero; or otherwise suitably transformed. Fixed effects modeled over the stride (i.e. those effects were not constant over a normalised stride) were stride percentage (0–100 %), and whether the nose angle increased or decreased. Fixed effects trial-level (effects that were constant over a normalised stride) variables were nose angle ROM (first tested as a dummy variable to check linearity versus rein tension), whether the horse-rider combination was turning (left/right or baseline not turning), passed through a corner (left/right or baseline not passing through corners), performed lateral movements (shoulder-in left/right direction, half-pass left/right direction, leg-yield left/right direction or baseline no lateral movements) or was riding in lengthening (only trot). The activity was also categorized according to position in saddle (sitting/rising to the trot). Horse level was included as a fixed effect. Left/right rein was forced in as a fixed effect. Random effects were horse-side, rider and horse and category within horse-side, the horse-side effect essentially modeling left/right reins in the random effect. The 2-way interaction between rein and stride percentage was tested. The percentage of the variation contributed by horse and rider was estimated, dividing by the sum of all sources of variation. Horse-specific models were also developed and in these the random effects were reduced to only trial within horse-side for the horse models, and fixed effects with single categories were successively removed. Models were reduced based on the type III sums of squares. The correlation structure was variance component. PROC MIXED (SAS Institute Inc., Cary, NC, 27513, USA) was used for modeling. Variables were retained if *P* < 0.05. In the graphs pair-wise comparisons were considered significant if *P* < 0.0001. Stride data were demonstrated by using the same type of modelled data for position in saddle and left/right turns.

## Results

### Descriptive data

**Fig. 1:**
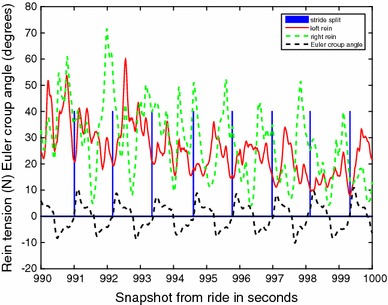
Graph of raw (calibrated) rein tension data at walk (rider 8, horse 1, riding straight). *Blue bars* indicate the stride split

**Fig. 2:**
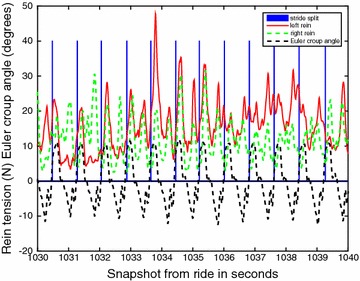
Graph of raw (calibrated) *left* and *right* rein tension data at rising trot (first straight and then turning *left*, rider 6, horse 1). *Blue bars* indicate the stride split

**Fig. 3:**
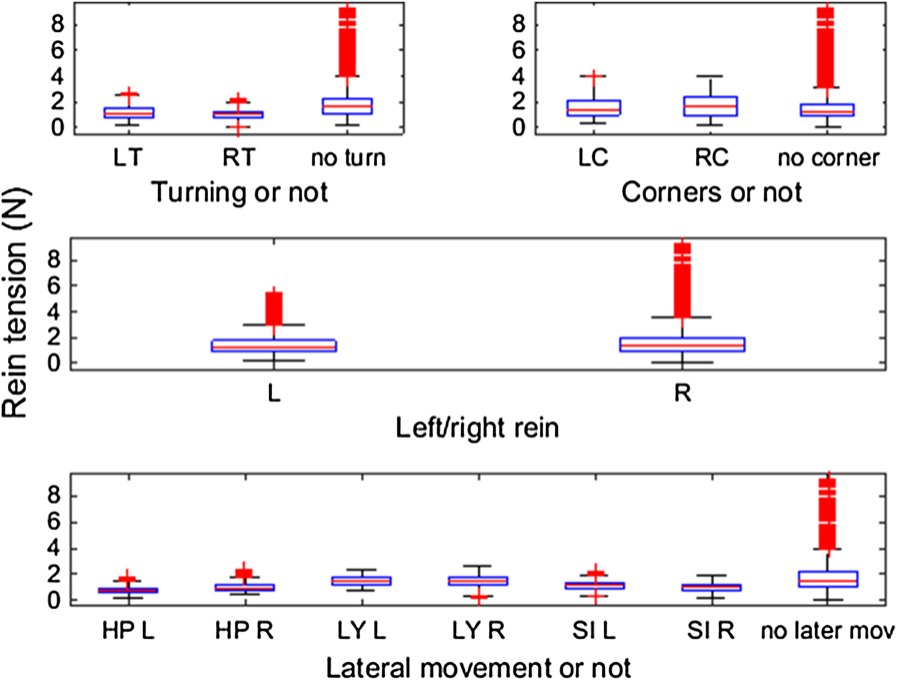
Boxplot of rein tension in walk by, turns (*LT* turn to the left, *RT* turn to the right and no turn), corners (*LC* corner to the left, *RC* corner to the right and no corner), *left* (L)/*right* (R) rein and lateral movements (HP/LY/SI Left/Right = half-pass/leg-yield/shoulder-into *left* and *right* direction in averaged stride split data from 6 riders and 18 horses, n = 21,008 data points)

**Fig. 4:**
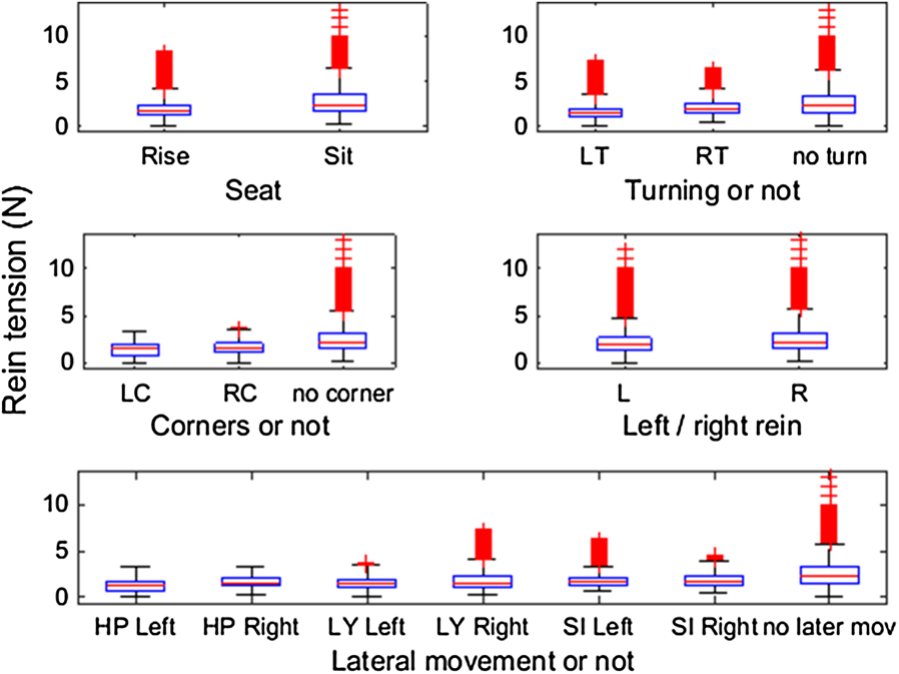
Boxplot of rein tension in trot by position in saddle (*Rise* rising trot, *Sit* sitting trot), turns (*LT* turn to the left, *RT* turn to the right and no turn), corners (*LC* corner to the left, *RC* corner to the right and no corner), left (L)/right (R) rein and lateral movements (HP/LY/SI Left/Right = half-pass/leg-yield/shoulder-into *left* and *right* direction in averaged stride split data from 6 riders and 18 horses, n = 37,168 data points)

The 18 horses ridden by the six riders were ridden during 1.5–19 min in walk on short reins and for a period of 4–19 min on short reins in trot. Three riders only used rising trot while trotting, while for the others the proportion of rising trot of all time trotted varied from 43 to 99 %, median 72 %. From these time slots in total 3118 walk strides and 9308 trot strides were selected after stride split. Within-horse, the number of strides per category of defined activity varied from 3 to 500. Figures 1 and 2 demonstrate a sample of raw data for walk and trot respectively, demonstrating the variation in the rein tension signal and the localisation of the stride split. Figures 3 and 4 demonstrate the distribution of the rein tension data by rein (left/right) for the variables turns, corners, position in saddle and lateral movements in walk and trot. Table 1 demonstrates descriptive statistics (rein tension and degrees) related to nose angle direction, nose angle ROM and lengthening in trot.

**Table 1:** Descriptive statistics (std- standard deviation; p5, p50, p95- 5th, 50th and 95 % percentiles) for nose direction in both walk and trot and lengthening in trot [rein tension (N)], nose angle range of motion (ROM; degrees)

Gait	Variable	Category (if relevant)	n	Rein tension (N)				
				Mean	std	p5	p50	p95
Walk	Nose direction	In	9906	15	8	5	13	29
		Out	11,102	14	8	5	13	29
Trot	Nose direction	In	16,998	23	14	7	19	50
		Out	20,170	25	16	8	21	56
	Lengthening		606	30	14	10	29	54

Gait	Variable	Category (if relevant)	n	Degrees				
				Mean	std	p5	p50	p95
Walk	Nose angle ROM		21,008	17	7	8	17	29
Trot	Nose angle ROM		37,168	8	4	4	8	16

Data on nose angle ROM are presented above and these were analysed multivariably in categorized formats (categories in walk see Table 2) and in trot; <5°, ≥5°–9°, ≥9°–13°, ≥13°–17° and ≥17°

### The models

Rein tension was deemed best as square root transformed in walk and best as logarithm transformed in trot. In walk the transformed distribution was as follows: mean 3.68; std 0.99; median 3.57; 5th percentile 2.24; 95th percentile 5.38; 21,008 observations/208 normalised strides (101 data points per normalised stride and rein). In trot the transformed distribution was as follows: mean 2.96; std 0.68; median 2.98; 5th percentile 1.92; 95th percentile 3.95; 37,168 observations/368 normalised strides. From the final walk model 29 %/27 % of the variation originated from the rider and horse respectively and these figures in trot were 20 %/7 %.

In the walk model the remaining variables were stride percentage (*P* < 0.0001), the interaction stride percentage and rein (*P* < 0.0001), whether the nose was moving in or out (*P* < 0.0001) and nose angle ROM (*P* = 0.01). Table 2 shows least square means that are controlled for each of the other variables in the model. The nose going out was associated with a higher value than if the nose was moving towards the horse (i.e. towards a position behind the vertical), and having a small head ROM (<12° and ≥12° < 16°) was associated with higher rein tension, compared to if the ROM was larger.

**Table 2:** Back-transformed least square means from the multivariable modeling of rein tension (N) in walk (data from 6 riders and 18 horses, n = 21,008 data points/208 normalised strides)

Variable	Category	LS mean	Group *P* value	Significant within-category		
Rein	Left	12.5	0.67			
	Right	12.8				
Nose direction	In	12.3	<0.0001	c		
	Out	12.9		c		
Nose angle ROM (degrees^a^)	<12	14.1	0.01	a	b	
	≥12–16	13.8		a		b
	≥16–20	11.8				
	≥20–24	12.9				
	≥24	10.6			b	b

The model also contained the fixed effects of stride percentage (*P* < 0.0001) and its interaction with rein (*P* < 0.0001). If pair-wise comparisons within a variable were associated with *P* < 0.0001 this is marked with ‘c’, if 0.01 < *P* ≥ 0.0001 then ‘b’ and if *P* < 0.01 then ‘a’, these letters are indicated in both categories

^a^ The nose angle categories contained from top to bottom: 4747; 3939; 6666; 3434 and 2222 observations. Rein tension was modelled square root transformed, hence confidence intervals could not be produced on the back-transformed scale

In the trot model nose angle ROM was not retained in the model, while all other variables remained (Table 3). Stride percentage (*P* < 0.0001) and the interaction stride percentage and rein were significant (*P* < 0.0001). Turns (*P* < 0.0001), corners (*P* = 0.0003) and lateral movements (*P* = 0.001) were associated with higher rein tension compared to their baselines. From Table 3 we note that none of the comparisons between specific exercises to the left and right (within corners, turns and lateral movements) were significant. Rising trot was associated with a lower rein tension compared to sitting trot (*P* < 0.0001). Lengthening the stride was associated with a higher rein tension than not lengthening (*P* = 0.01). Also moving out (i.e. towards a position in front of the vertical) of the nose in trot was associated with a higher rein tension than moving inwards (*P* < 0.0001). Horse level was significant (*P* = 0.01). Rein tension decreased among horse categories in the following order: advanced horses/young horses/medium and basic horses, with several significant pairwise comparisons.

**Table 3:** Back-transformed least square means (LS means) for rein tension in trot (logarithm transformation, in N) from multivariable modeling (data from 6 riders and 18 horses, n = 37,168 data points/368 normalised strides)

Variable	Category	LS mean 95 % CI	Group	Significant within-category			
			*P* value				
Turn	Turn L	37 (27, 51)	<0.0001	c			
	Turn R	29 (22, 38)			b		
	BL	36 (26, 50)		c	b		
Corner	Corner L	39 (28, 54)	0.0003	c			
	Corner R	34 (24, 48)			b		
	BL	29 (22, 38)		c	b		
Lateral movements	Leg-yield L	28 (21, 37)	0.001	a			
	Leg-yield R	33 (23, 46)			a		
	Half-pass L	33 (23, 46)					
	Half-pass R	32 (22, 45)				b	
	Shoulder-in L	40 (28, 58)					b
	Shoulder-in R	38 (27, 53)					
	BL	34 (24, 47)		a	a	b	b
Seat	Rising	28 (21, 39)	<0.0001	c			
	Sitting	40 (29, 54)		c			
Lengthening	Lengthening	28 (22, 36)	0.01	a			
	BL	40 (27, 61)		a			
Nose direction	In	32 (23, 43)	0.0001	b			
	Out	36 (26, 49)		b			
Rein	Left	33 (25, 45)	0.01	a			
	Right	34 (25, 46)		a			
Horse level	Advanced	45 (30, 68)	0.01	a	b		
	Medium	28 (20, 41)		a			
	Young horse	38 (26, 56)				b	
	Basic	26 (19, 37)			b	b	

The model also contained the fixed effects of stride percentage (*P* < 0.0001) and its interaction with rein (*P* < 0.0001). If pair-wise comparisons within a variable were associated with *P* < 0.0001 this is marked with ‘c’, if 0.01 < *P* ≥ 0.0001 then ‘b’ and if *P* < 0.01 then ‘a’, these letters are indicated in both categories. For lateral movements only comparisons to baseline (B) and within the same type of movements were performed (e.g. half-pass left (L) and right (R) were not significantly different and the comparison is therefore not shown)

**Fig. 5:**
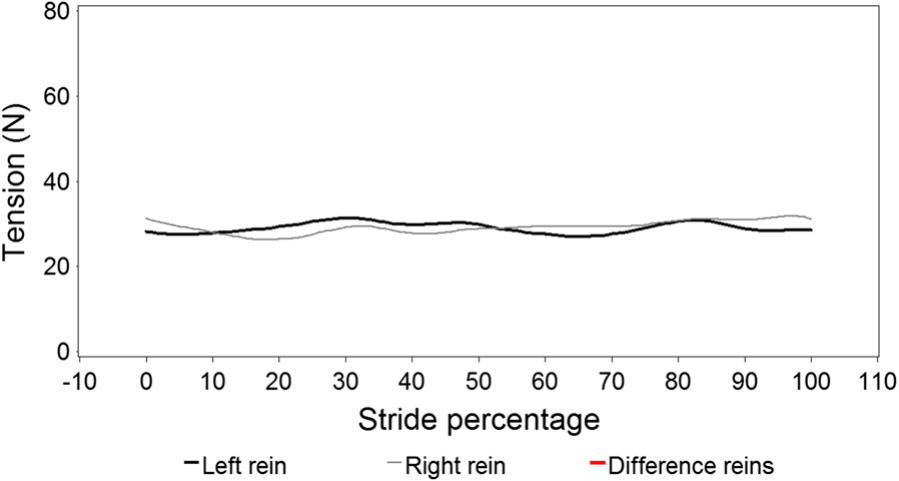
Modelled rein tension in walk during an average walk stride, controlling for the variables found in Table 2. Stride percentages zero and 100 represent mid-stance of the right forelimb. There are no significant differences between the *left* and *right* rein (no *red line* sections)

**Fig. 6:**
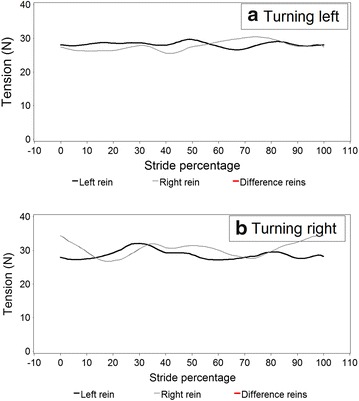
Modelled rein tension in walk during a walk stride based on data where the horse was ridden in turns, **a** turning to the *left* and **b** to the *right*. Stride percentages zero and 100 represent mid-stance of the *right* forelimb. There are no significant differences between the *left* and *right* rein (no *red line* sections)

**Fig. 7:**
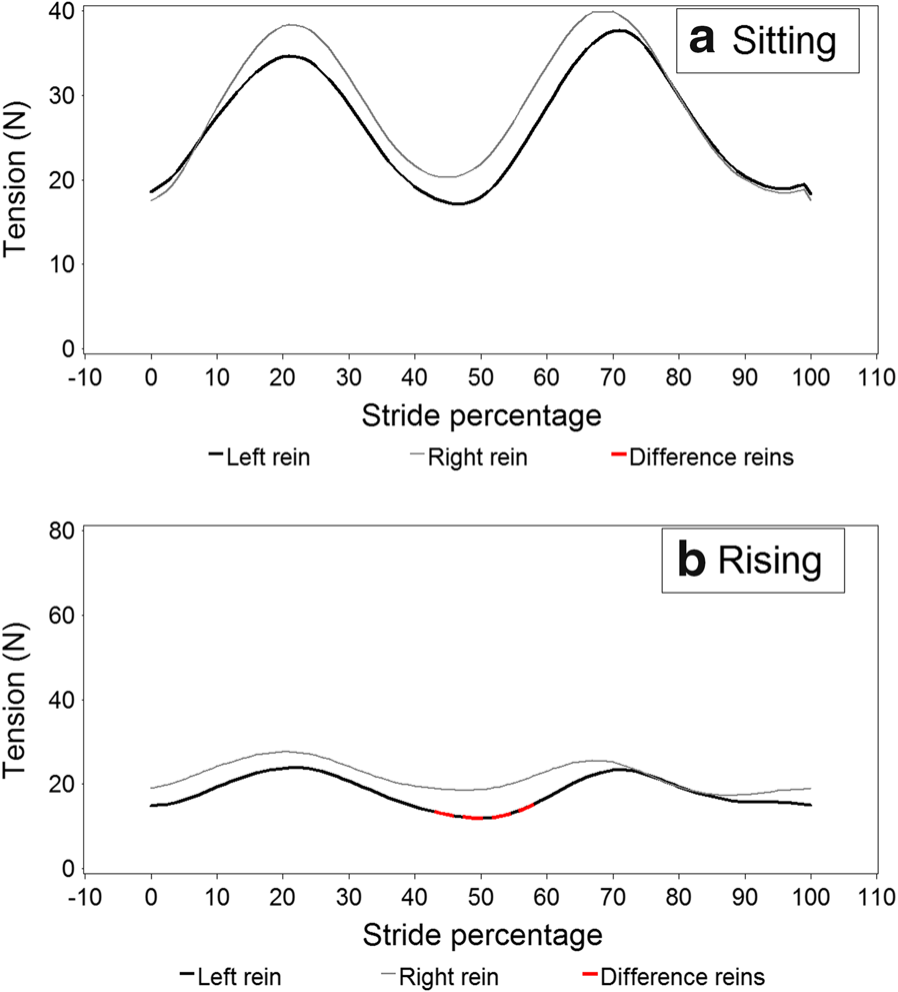
Schematic rein tension in trot on the *left* and *right* reins (rein L and rein R) during a stride based on data where the horse was ridden in **a** sitting trot (in the model; 6 riders, 15 horses, 16,968 data points) compared to **b** rising trot (6 riders, 18 horses, 20,198 data points), Stride percentages zero and 100 represent mid-stance of the *right* forelimb. *Red coloured line* sections in the *left* rein demonstrates significant differences between the *left* and *right* rein (note that scales are different)

**Fig. 8:**
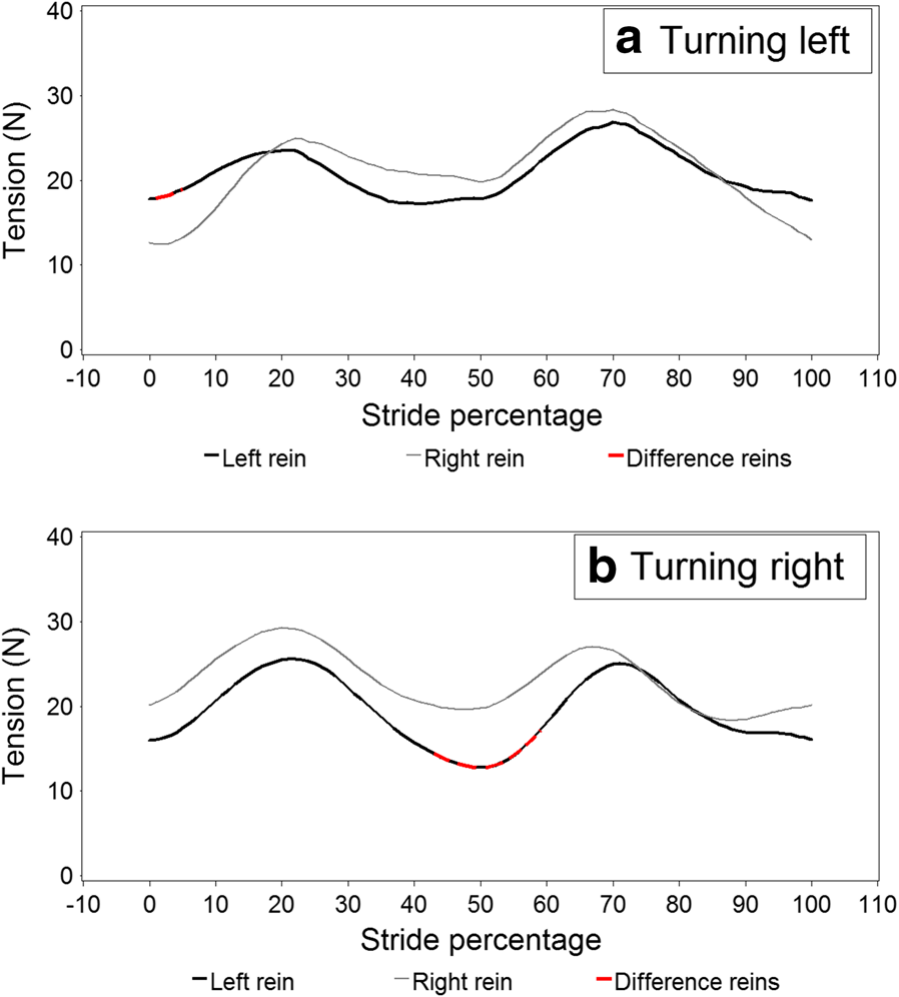
Schematic rein tension in trot in the *left* and *right* reins (rein L and rein R) during a stride based on data where the horse was ridden turning **a** to the left (in the model; 6 riders, 18 horses, 7474 data points) and **b** to the right based on modelled data (6 riders, 18 horses, 6868 data points). Stride percentages zero and 100 represent mid-stance of the right forelimb. *Red coloured line* sections in the *left* rein demonstrate significant differences between the *left* and *right* rein

Figures 5 and 6 illustrate modelled stride curves for walk overall and for turning left and right. A significantly higher rein tension in the right rein at 50 % of the stride was recorded during rising trot compared to sitting trot (Fig. 7). Given the model, this interaction was controlled for bend orientation of the horse and direction of travel. Comparing sitting to rising trot, in rising trot significantly higher rein tension in the right rein was found at 50 % of the stride (Fig. 7). The inside rein (the rein facing the inside of an arena or a circle) has higher tension than the outside rein when the horse is turning, both left and right, with the horse on the outer fore-inner hind limb diagonal (Fig. 8).

A biphasic pattern corresponding with the two diagonal phases of the trot was found in all 18 horses, and most often for both reins (Additional file 3, Additional file 4). The maxima were most commonly found at around 10–30 and 60–80 % of the stride, coinciding with the suspension phase. One rider demonstrated a biphasic pattern in the walk (rider 4 in 2 horses) with the left rein showing a more pronounced biphasic pattern compared to the right rein, which showed a flatter signal and there were also statistically significant differences between the reins in this rider (statistically significant differences in walk were found in two riders and four horses). At trot, riders 4, 6 and 7 presented with significant, but non-systematic, within-horse differences between the left and right rein. These differences were non-systematic, as they were not found in the same parts of the stride cycle; some differences were found closer to the minimum rein tension and some closer to the maximum rein tension. For example in rider 4, horse 1 in trot (Additional file 4) the left rein has significantly higher rein tension around 5–20 % of the stride and in horse 3 the right rein is associated with the highest rein tension, but around 30–50 and 75–100 % of the stride.

## Discussion

### Between-gait, rider and horse variation

Both rider and horse variations were substantial at walk, while rider variation was relatively higher than horse variation at trot. This may have to do with the very regular character of the latter gait, which is characterized by low ranges of motion of the equine thoracolumbar column [16]. In trot the pattern in each rider was similar but the significant differences that were seen were not consistently found in the same phases of the stride cycle. Additional files 3 and 4 also suggest within-rider differences, both related to timing and the level of rein tension.

### Reins, turns and position within saddle

Figure 5 and Additional file 3 demonstrate that the average pattern in walk does not have a biphasic nature. The graphs for turning left and right in walk (Fig. 6) suggest a phase shift between the reins. The data were stride split on right forelimb midstance and turning right the left rein had its maxima at 30 and 80 %, which is at left and right hindlimb midstance, while the right rein had the maxima at 95 and 35 %, at the beginning of right and left forelimb stance. At turning left, the maxima of the left rein were at 50 and 85 % (left forelimb midstance and initiation of right forelimb stance), and those of the right rein at 35 and 70 % (left and right hindlimb midstance). The between-graph differences are likely to be due to the fact that the various individual differences preclude a stable common pattern, but the reasons for the individual differences (as reflected by the large shares of rider and horse variation) are likely locomotory habits and laterality issues in the riders and the horses. Several differences between the two reins have been demonstrated [8]. The authors’ interpretation of these findings was that this is most likely related to laterality in horses and riders, but this cannot be substantiated based on the current data.

In trot the right rein is associated with slightly higher rein tension, which was shown to be significant in rising trot between 42 and 58 % of the stride, i.e. during left forelimb stance. The riders actually managed to ride during equal time periods rising trot in left and right directions, and were in general rising to the ‘recommended’ diagonal, namely rising on the inside forelimb, outside hindlimb [17]. At the trot, when the horses were turning left and right respectively and when the horses were using the outer fore- inner hindlimb diagonal the inner rein was acting more intensively. This was likely because the rider strived at taking the turn with the longitudinal axis of the horse in a regularly bent position. It is possible that riders were applying greater tension on the inner rein in order to achieve slight lateral bending of the horses’ head in the direction of the turn, following standard recommendations from riding manuals [17]. Additionally, turns and corners to the right were associated with lower rein tension than turns and corners to the left and the general baseline (Table 3), which is likely a function of rider- and or horse laterality. Ideally, the rider should be able to manage both the rider’s laterality and that of the horse to the degree that right and left corners would be associated with the same rein tension levels. Previous research has demonstrated that this may not always be the case [8]. Left rein tension has been found to be more stable and generally higher than the tension in the right rein when subjects simulated halts on a model horse. This was interpreted as being in agreement with the fact that the left hand/rein acts more based on *postural input*, while the actions and reactions of the right hand, featuring both high and low tension spikes, is more dependent on *visual input* [19].

### The results from the multivariable models

At walk, in general nose angle ROM had lower values when rein tension was higher (Table 2). Rein tension was likely lower when horses were walked in a less restrained (restraining being normally effectuated by being held back by the rider or moving slowly) way, i.e. principally at higher speed and with a greater pendulum movement of the head. Nose angle ROM was not significant in trot, which is possibly caused by the more limited ROM of the head in this gait that is, as alluded to earlier, characterized by a low range of motion of all parts of the axial skeleton [16], including the cervical part and thus the head that is attached to this.

Rein tension was higher when the nose was moving forwards or ‘out’ (moving towards a position in front of the vertical) in relation to the body in both gaits (in both gaits this is from 0 to 25 and 50 to 75 % of the stride, data not shown). Either the rider was not ‘following’ as well as they intended (because of the motion of the head of the horse the riders need to adjust hand-position in every stride), or the riders were actively trying to interact, either trying to bring the horse’s head closer to the chest or trying to make it slow down and/or increase collection. In that latter case the cue might not have been working well, as the tension was found to be significantly higher when the nose was moving forwards. While riding in trot, the maximal distance from the riders hand to the mouth of the horse is found at midstance [10], which perhaps indicates that ‘following’ the horse’s movement by the rider is difficult. Terada et al. [18] investigated the timing of activity of rider muscles and found several muscles that were activated during early stance, including the *m. biceps brachii* and the middle deltoid muscle. This may demonstrate a ‘taking’ mechanism that may either be an active choice, that is the rider is aware of doing it and can choose to perform it or not, or perhaps a more reflex-based activity that may be a posturally elicited mechanism that the rider is more unaware of. The various lateral movements all led to rein tensions differing from baselines. Magnitude and direction of these changes were variable, however, and require additional work to be fully understood. As found previously, sitting trot and lengthening were associated with higher rein tension than their counterparts, rising and no lengthening. The reason is likely that more difficult exercises are performed when sitting/lengthening. Sitting trot and riding a lengthened stride likely means that the rider’s body is experiencing a more violent acceleration/deceleration pattern so the increased tension may be an unconscious (or semi-automated) postural anti acceleration response. With regard to horse level advanced and young horses were ridden with higher rein tension, which was almost double compared to basic horses. This may be because both of these groups are asked to perform either absolutely or relatively (given the level of training) difficult and demanding exercises.

### Limitations of the study

Since there was a problem with the synchronization of the signals form horse’s croup and head in two riders/six horses of the original study population [13], only six riders and 18 horses were included in the data analysis. Despite the set-up with one recording person and riding ‘as usual’ it is possible that riders were somewhat less relaxed compared to a typical training day, which may have influenced the results. Also, no strides featuring collection could be selected from the walk and trot data [20]. This was likely to be because the acceleration signals became more irregular during collection, precluding optimal stride split from the acceleration signal alone. Furthermore, the discussion on laterality in riders or horses was not based on measurements, but solely on subjective declarations by the riders.

## Conclusions

The gait the horse is ridden in has a clear influence on the rein tension pattern. Biphasic patterns were found mainly in trot. The highest rein tension was found in the suspension phase in trot, and the lowest in the stance phase. In walk, the highest rein tension was found at hindlimb stance. Studies of rein tension should hence take both the gait and the stride cycle phase into account. Further, there was substantial between-rider variation in walk and trot and between-horse variation in walk. These differences are most likely due to the much lower range of motion of the thoracic and lumbar equine vertebral column in trot compared to in walk. Future studies on rein tension should include analyses of detailed temporal relationships with horse kinematics, rider kinematics including the seat (taking into account the rider’s posture and relative positioning of all body parts), and of the behavior of the horse.

## Additional files

10.1186/s13028-015-0182-3 The rein tension meter used in the study. Earlier published in Eisersiö M, Roepstorff L, Rhodin M, Egenvall A. Rein tension in eight professional riders during regular training sessions. JVB: Clinical applications and research. 2015;10:419-426.
10.1186/s13028-015-0182-3 The graph shows an example where one meter that was tested with increasing tension up to 500 N (several cycles). The curve is almost linear, though slightly upward bent (both offset and this bend are corrected for in the calibration). The hysteresis effect was maximally 8 N, measured as the vertical distance between the lines.
10.1186/s13028-015-0182-3 Rein tension during the stride cycle at the walk for the left (black) and right (grey) rein per horse. Each row indicates one rider. Significant differences (P < 0.0001) between the left and right rein are shown as broken red lines in the inside rein. Stride percentages zero and 100 represent mid-stance of the right forelimb.
10.1186/s13028-015-0182-3 Rein tension during the stride cycle at the trot for the left (black) and right (grey) rein per horse. Each row indicates one rider. Significant differences (P < 0.0001) between the left and right rein are shown as broken red lines in the inside rein. Stride percentages zero and 100 represent mid-stance of the right forelimb.
