# Supplementary figures and images for: Stride-related rein tension patterns in walk and trot in the ridden horse
Source: Acta Vet Scand. 2015 Dec 30;57:89. doi: 10.1186/s13028-015-0182-3 (PMC4696263; doi:10.1186/s13028-015-0182-3)

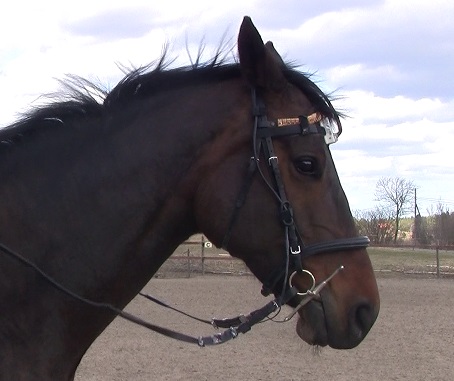

Supplement: Supplementary file 1 — 10.1186/s13028-015-0182-3 The rein tension meter used in the study. Earlier published in Eisersiö M, Roepstorff L, Rhodin M, Egenvall A. Rein tension in eight professional riders during regular training sessions. JVB: Clinical applications and research. 2015;10:419-426. [file 13028_2015_182_MOESM1_ESM.zip › vlcsnap-2013-05-07-20h25m38s148 Malva 2.jpg]

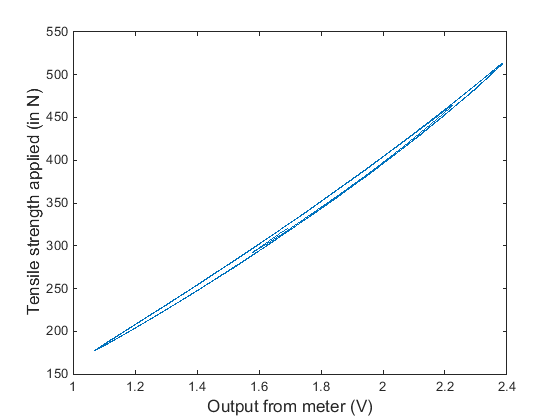

Supplement: Supplementary file 2 — 10.1186/s13028-015-0182-3 The graph shows an example where one meter that was tested with increasing tension up to 500 N (several cycles). The curve is almost linear, though slightly upward bent (both offset and this bend are corrected for in the calibration). The hysteresis effect was maximally 8 N, measured as the vertical distance between the lines. [file 13028_2015_182_MOESM2_ESM.tif]

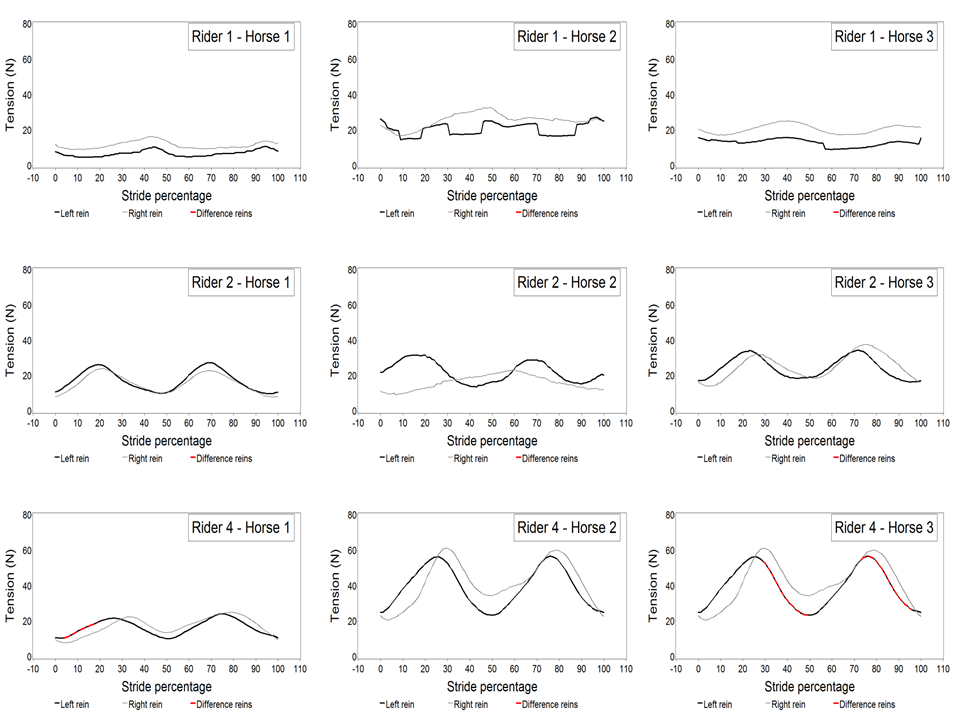

Supplement: Supplementary file 3 — 10.1186/s13028-015-0182-3 Rein tension during the stride cycle at the walk for the left (black) and right (grey) rein per horse. Each row indicates one rider. Significant differences (P < 0.0001) between the left and right rein are shown as broken red lines in the inside rein. Stride percentages zero and 100 represent mid-stance of the right forelimb. [file 13028_2015_182_MOESM3_ESM.zip › Slide1.TIF]

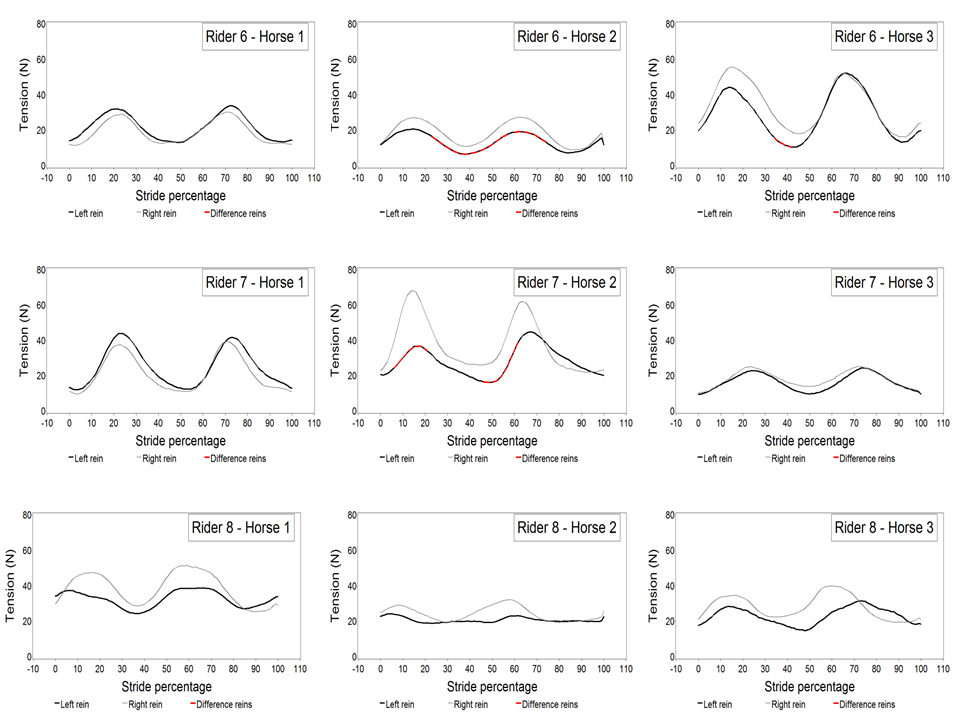

Supplement: Supplementary file 3 — 10.1186/s13028-015-0182-3 Rein tension during the stride cycle at the walk for the left (black) and right (grey) rein per horse. Each row indicates one rider. Significant differences (P < 0.0001) between the left and right rein are shown as broken red lines in the inside rein. Stride percentages zero and 100 represent mid-stance of the right forelimb. [file 13028_2015_182_MOESM3_ESM.zip › Slide2.TIF]

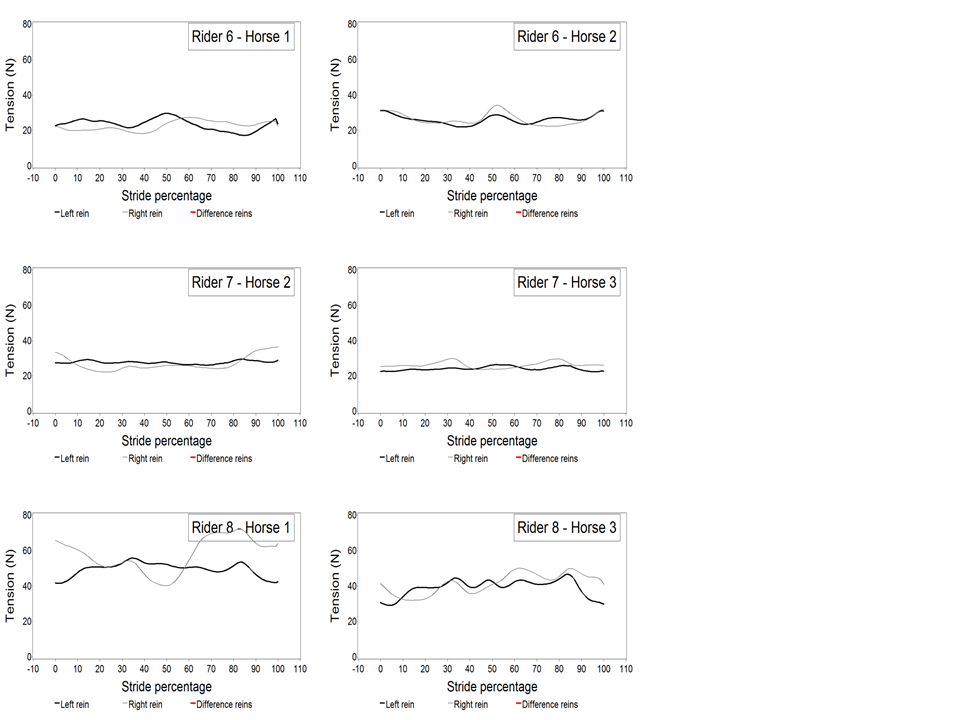

Supplement: Supplementary file 4 — 10.1186/s13028-015-0182-3 Rein tension during the stride cycle at the trot for the left (black) and right (grey) rein per horse. Each row indicates one rider. Significant differences (P < 0.0001) between the left and right rein are shown as broken red lines in the inside rein. Stride percentages zero and 100 represent mid-stance of the right forelimb. [file 13028_2015_182_MOESM4_ESM.zip › Slide4.TIF]

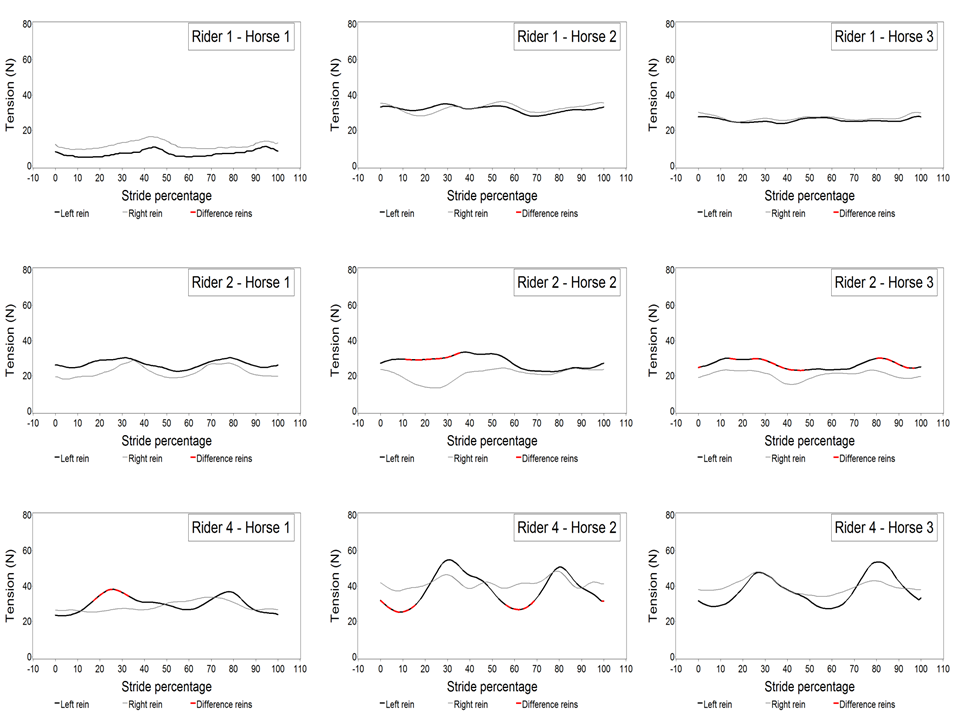

Supplement: Supplementary file 4 — 10.1186/s13028-015-0182-3 Rein tension during the stride cycle at the trot for the left (black) and right (grey) rein per horse. Each row indicates one rider. Significant differences (P < 0.0001) between the left and right rein are shown as broken red lines in the inside rein. Stride percentages zero and 100 represent mid-stance of the right forelimb. [file 13028_2015_182_MOESM4_ESM.zip › Slide3.TIF]
